# Properties of Transport Mediated by the Human Organic Cation Transporter 2 Studied in a Polarized Three-Dimensional Epithelial Cell Culture Model

**DOI:** 10.3390/ijms22179658

**Published:** 2021-09-06

**Authors:** Tim N. Koepp, Alexander Tokaj, Pavel I. Nedvetsky, Ana Carolina Conchon Costa, Beatrice Snieder, Rita Schröter, Giuliano Ciarimboli

**Affiliations:** Medicine Clinic D, Experimental Nephrology, University Hospital of Münster, 48149 Münster, Germany; tim.koepp123@gmail.com (T.N.K.); alex.tokaj@uni-muenster.de (A.T.); nedvetsky@uni-muenster.de (P.I.N.); carolconchon@gmail.com (A.C.C.C.); bea.snieder@gmail.com (B.S.); ritas@uni-muenster.de (R.S.)

**Keywords:** transport, organic cations, kidneys, cell polarization

## Abstract

The renal secretory clearance for organic cations (neurotransmitters, metabolism products and drugs) is mediated by transporters specifically expressed in the basolateral and apical plasma membrane domains of proximal tubule cells. Here, human organic cation transporter 2 (hOCT2) is the main transporter for organic cations in the basolateral membrane domain. In this study, we stably expressed hOCT2 in Madin-Darby Canine Kidney (MDCK) cells and cultivated these cells in the presence of an extracellular matrix to obtain three-dimensional (3D) structures (cysts). The transport properties of hOCT2 expressed in MDCK cysts were compared with those measured using human embryonic kidney cells (HEK293) stably transfected with hOCT2 (hOCT2-HEK cells). In the MDCK cysts, hOCT2 was expressed in the basolateral membrane domain and showed a significant uptake of the fluorescent organic cation 4-(4-(dimethylamino)styryl)-*N*-methylpyridinium (ASP^+^) with an affinity (K_m_) of 3.6 ± 1.2 µM, similar to what was measured in the hOCT2-HEK cells (K_m_ = 3.1 ± 0.2 µM). ASP^+^ uptake was inhibited by tetraethylammonium (TEA^+^), tetrapentylammonium (TPA^+^), metformin and baricitinib both in the hOCT2-HEK cells and the hOCT2- MDCK cysts, even though the apparent affinities of TEA^+^ and baricitinib were dependent on the expression system. Then, hOCT2 was subjected to the same rapid regulation by inhibition of p56*^lck^* tyrosine kinase or calmodulin in the hOCT2-HEK cells and hOCT2- MDCK cysts. However, inhibition of casein kinase II regulated only activity of hOCT2 expressed in MDCK cysts and not in HEK cells. Taken together, these results suggest that the 3D cell culture model is a suitable tool for the functional analysis of hOCT2 transport properties, depending on cell polarization.

## 1. Introduction

Human organic cation transporter 2 (hOCT2) is highly expressed on the basolateral membrane domain (facing the interstitial space) of renal proximal tubule cells [1,2,3]. Here, hOCT2 transports organic cations (OCs) across the plasma membrane according to their electrochemical gradients [4,5]. Organic cations are then excreted from the cells of proximal tubules into the urine by apically expressed (facing the urine) transporters such as the Multidrug and Toxin Extrusion (MATE) proteins, which function as H^+^/OC exchangers [3,6]. Together, these transporters build a vectorial transporter system, which determines the renal secretory clearance of an OC, a pivotal renal function [7]. Many important physiological and pharmacological substances such as neurotransmitters (e.g., dopamine and serotonin) [8,9], metabolism products like creatinine and spermidine [10,11,12], antidiabetic drugs (e.g., metformin) [13,14], antiarrhythmic drugs (e.g., procainamide) [15] and ß-blockers (e.g., atenolol) [16,17,18] are OCs. Therefore, hOCT2 functioning may have important physiological and pharmacological implications.

Relevant information about hOCT2 functional properties, regulation and interaction with drugs has been obtained using expression systems such as human embryonic kidney cells (HEK293) (see, for example, [19,20]) and *Xenopus* oocytes (see, for example, [21,22,23]). However, these cellular systems do not have distinct apical and basolateral membrane domains, the formation of which is essential for proper function of the polarized renal tubule cells, where these transporters are abundantly expressed and functionally important. Therefore, in this work, we established a three-dimensional (3D) cell model of highly polarized Madin Darby Canine Kidney (MDCK) cells expressing hOCT2. For this purpose, MDCK cells stably expressing hOCT2 were grown in the presence of an extracellular matrix, which led to the formation of spherical cysts with a hollow lumen surrounded by a single layer of polarized cells [24]. The hOCT2 expressed in MDCK cysts was localized at the basolateral membrane and easily accessible for transport studies. Therefore, the functional properties of hOCT2 stably expressed in MDCK cysts and HEK293 cells were compared to study whether and how a polarized expression system could influence hOCT2 activity.

## 2. Results

### 2.1. Characterization of MDCK Cysts

**Figure 1:**
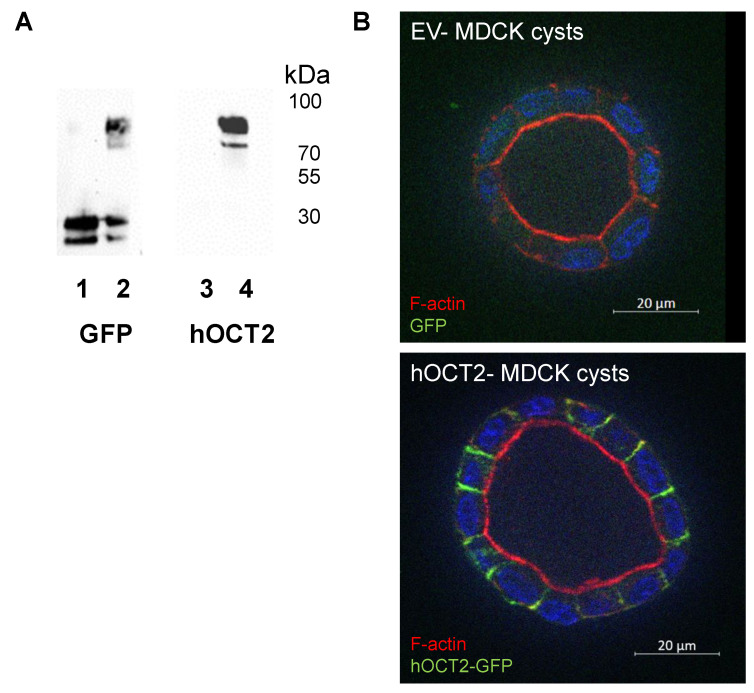
Characterization of MDCK cysts. Panel (**A**) shows a western blot analysis of lysates from the MDCK cells stably expressing the EV (lanes 1 and 3) or hOCT2-GFP (lanes 2 and 4) constructs. The membranes were incubated with antibodies against GFP (lanes 1 and 2) or against hOCT2 (lanes 3 and 4). Cells expressing EV displayed two bands below the 30 kDa molecular weight marker under GFP detection, probably corresponding to GFP alone and its degradation product (lane 1). The labeling for hOCT2 in EV-expressing cells did not show any signal at the hOCT2 molecular weight (lane 3). Cells expressing hOCT2-GFP displayed two bands between 100 and 70 kDa under GFP detection, probably corresponding to glycosylated and non-glycosylated hOCT2-GFP (lane 2). Staining for hOCT2 revealed the same bands detected with an anti-GFP antibody between 100 and 70 kDa (lane 4), indicating that these cells expressed the hOCT2-GFP construct. Panel (**B**) shows an immunofluorescence analysis of the EV (upper panel) and hOCT2-GFP (lower panel) expressing MDCK cells growing in BME2 gel. These cells built cysts with a distinct basolateral and apical membrane domain. F-actin staining showed the apical membrane in red in both preparations. Only in hOCT2-GFP-expressing cysts was a clear GFP signal observed at the basolateral plasma membrane domain (green color, lower panel). Scale bar = 20 µm.

As shown by western blot analysis, the viral transduction of MDCK cells with GFP (empty vector (EV)) resulted in the expression of GFP (lane 1 in Figure 1A). In these cells, when using an antibody against hOCT2, no specific signal for hOCT2 was detected (lane 3 in Figure 1A). In MDCK cells transduced with hOCT2-GFP, bands corresponding to the hOCT2 transporter tagged with GFP were detected both using antibodies against GFP or hOCT2 (lanes 2 and 4 in Figure 1A, respectively). When cultured in a basement membrane extract (BME2) gel, MDCK cells formed well-polarized cysts, with clearly separated apical and basolateral membrane domains. Labeling of F-actin clearly showed the apical membrane domain (Figure 1B), as it is well known for MDCK cysts (see, for example [25]). GFP fluorescence was clearly localized to the basolateral membrane domain only in MDCK cells expressing hOCT2-GFP (Figure 1B, lower panel).

### 2.2. Uptake of the Fluorescent Organic Cation 4-(4-(Dimethylamino)styryl)-N-methylpyridinium (ASP^+^) in MDCK Cysts

**Figure 2:**
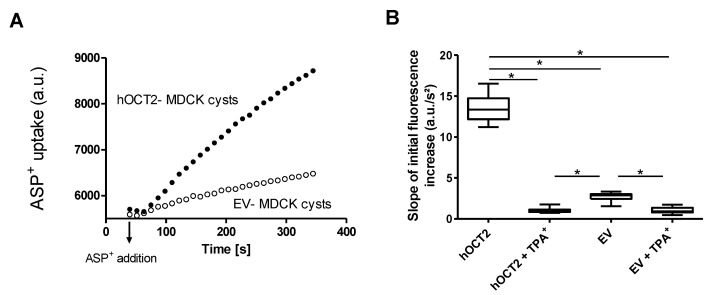
Uptake of ASP^+^ in MDCK cysts. Panel (**A**) shows an example of the ASP^+^ uptake experiments in hOCT2-MDCK and EV-MDCK cysts. Shortly after the addition of 4 µM ASP^+^, an increase of cellular fluorescence (measured as arbitrary units (a.u.)) over time was observed. This increase was linear in the first 2 min (initial uptake) and was stronger in MDCK cysts expressing hOCT2-GFP (closed dots) than in cysts expressing GFP alone (EV, open dots). Panel (**B**) shows the slope of the initial ASP^+^ uptake increase measured in a.u./s^2^ in hOCT2-MDCK (hOCT2)- and EV-MDCK (EV) cysts in the presence or lack of 1 mM TPA^+^ as a competitor for OCT-mediated transport (*N* = 3 independent experiments, *n* = 9 (3 replicates/experiment)). Data are shown as box and whisker plots (min to max). The stars show statistically significant differences (* *p* < 0.05, Anova with Tukey’s multiple comparison test).

Figure 2A shows an example of an ASP^+^ uptake experiment in hOCT2-MDCK and EV-MDCK cysts. Upon the addition of 4 µM ASP^+^, an increase in cellular fluorescence was observed over time. This fluorescence increase was much higher in the hOCT2-MDCK than the EV-MDCK cysts. Still, in the EV-MDCK cysts, a small ASP^+^ uptake was also observed, probably due to an endogenous expression of OCT2 in MDCK cells, as suggested by Ludwig et al. [26]. The slope of the linear range of the ASP^+^ uptake increase measured in the first 2 min after ASP^+^ addition (arbitrary fluorescence units/s^2^) was used as the measurement of the transporter function.

As shown in Figure 2B, the uptake of 4 µM ASP^+^ in the hOCT2 cysts (hOCT2) was almost completely inhibited by the simultaneous treatment with a high tetrapentylammonium (TPA^+^, a potent inhibitor of OCT [21,27]) concentration (1 mM) in the uptake buffer. Even though the ASP^+^ uptake in the GFP cysts (EV) was much smaller, it was still significantly inhibited by 1 mM TPA^+^.

### 2.3. Comparison of ASP^+^ Transport Mediated by hOCT2 Stably Expressed in HEK293 (hOCT2-HEK) Cells or in MDCK Cysts (hOCT2- MDCK)

**Figure 3:**
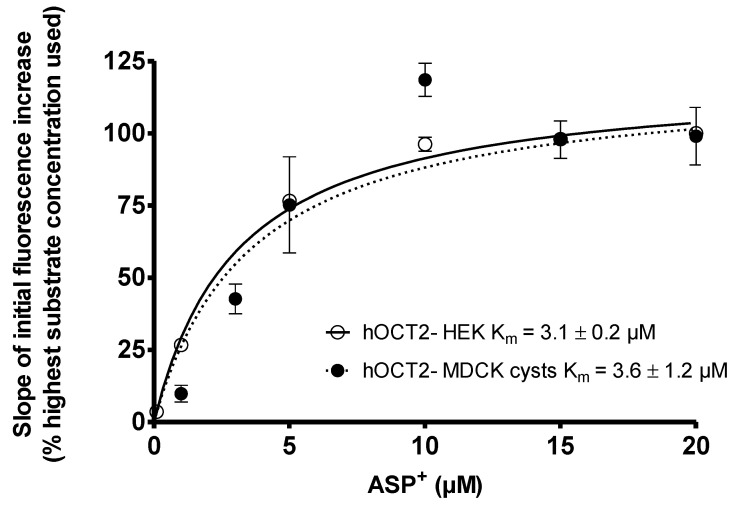
Specific ASP^+^ uptake mediated by hOCT2 stably expressed in HEK cells (hOCT2-HEK, open dots) or in MDCK cysts (hOCT2-MDCK cysts, closed dots) by adding rising concentrations of ASP^+^ to the cells and detecting the fluorescence intensity. The specific uptake was measured by subtracting the unspecific uptake determined in the presence of 1 mM TPA^+^ from the total uptake. Since the absolute fluorescence values are very different in hOCT2-HEK cells and hOCT2-MDCK cysts, to be able to make the two curves visible in the same graphic, the data were normalized to the maximal uptake measured in each model system with 20 µM ASP^+^, which was set to 100%. Data are shown as the mean ± SEM, calculated from at least three replicates/ASP^+^ concentration measured in at least three independent experiments. In the insert, the calculated K_m_ values for the two expression systems are shown.

First, an ASP^+^ saturation curve for the hOCT2-HEK cells and hOCT2- MDCK cysts was measured. To calculate the hOCT2-specific ASP^+^ uptake, the transport measured in the presence of 1 mM TPA^+^ was subtracted from the total ASP^+^ uptake for every ASP^+^ concentration tested (not shown). Since the absolute values of the uptake measured in the hOCT2-HEK cells and hOCT2- MDCK cysts were rather different, the values were normalized as percentages of the maximal fluorescence reached for every saturation curve. Of course, using this method, a comparison of the V_max_ values between two cell lines was not possible. However, the V_max_ values were dependent upon the expression rate, which could be different in the two cell preparations. As shown in Figure 3, the specific ASP^+^ uptake reached a saturation at 20 µM both in the hOCT2-HEK cells and the hOCT2-MDCK cysts. The saturation curves measured for the two different expression models were almost superimposable, resulting in very similar K_m_ values (mean ± SEM) of 3.1 ± 0.2 and 3.6 ± 1.2 µM for the hOCT2-HEK cells and hOCT2- MDCK cysts, respectively.

**Figure 4:**
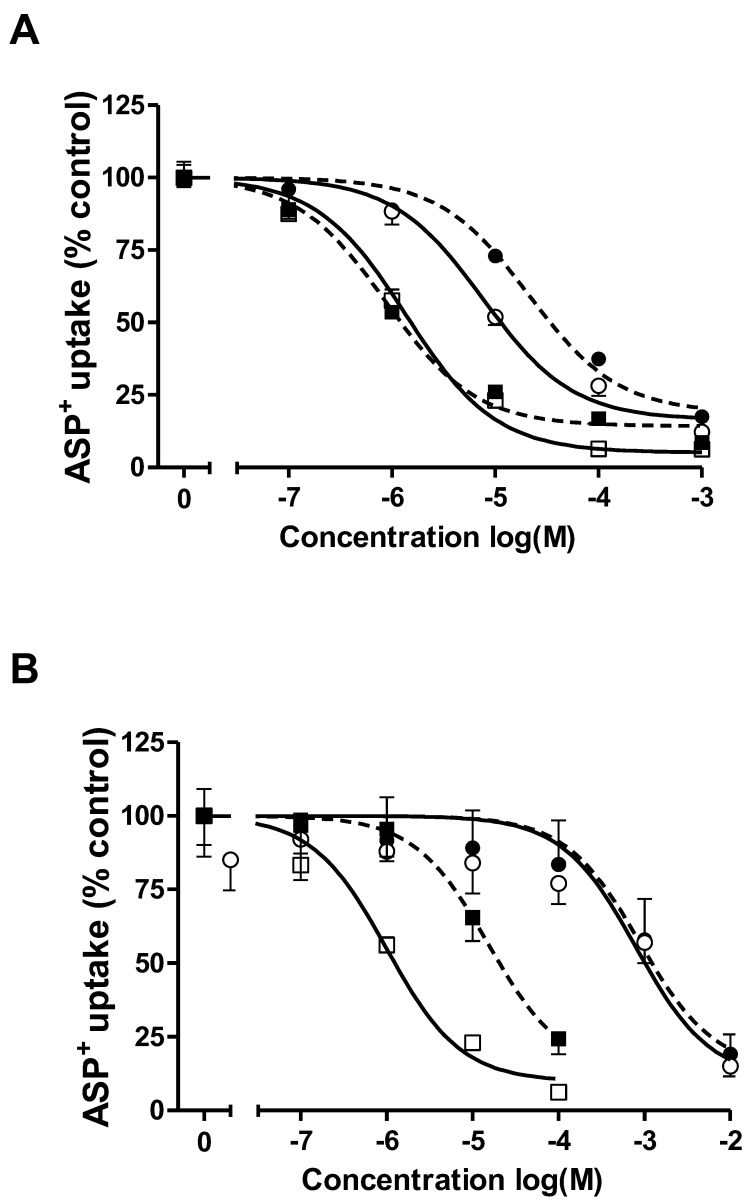
Concentration response curves for the inhibition of initial ASP^+^ uptake, mediated by hOCT2 stably expressed in HEK cells (hOCT2-HEK, open symbols) or in MDCK cysts (hOCT2-MDCK cysts, (closed symbols)) by the organic cations TPA^+^ (squares) and TEA^+^ (dots) (panel **A**) and by the drugs baricitinib (squares) and metformin (dots) (panel **B**). Values are the means ± SEM calculated from at least three independent experiments and expressed as percentages of ASP^+^ uptake in the absence of the inhibitor.

The apparent affinities of the hOCT2-HEK and hOCT2-MDCK cysts for substances known to compete with ASP^+^ transport by hOCT2 (tetraethylammonium (TEA^+^), TPA^+^, metformin and baricitinib) are shown in Figure 4 and summarized in Table 1. The IC_50_ values for TPA^+^ inhibition of ASP^+^ uptake were very similar in the hOCT2-HEK cells (Figure 4A, open squares; 1.4 µM, log IC_50_ = −5.9 ± 0.2) and hOCT2-MDCK cysts (Figure 4A, closed squares; 0.9 µM, log IC_50_ = −6.1 ± 0.1). Conversely, the IC_50_ values for TEA^+^ inhibition were significantly different (Figure 4A, open dots for hOCT2-HEK cells: 7.6 µM, log IC_50_ = −5.1 ± 0.1; closed dots for hOCT2-MDCK cysts: 21.3 µM, log IC_50_ = −4.7 ± 0.1). The IC_50_ values for metformin inhibition of the ASP^+^ uptake were very similar in the hOCT2-HEK cells and hOCT2-MDCK cysts (804 µM, log IC_50_ = −3.1 ± 0.1 in hOCT2-HEK cells, open dots and 884 µM, log IC_50_ = −3.1 ± 0.1 in hOCT2-MDCK cysts, closed dots, Figure 4B). Conversely, the IC_50_ values for baricitinib inhibition were significantly different (1.1 µM, log IC_50_ = −6.0 ± 0.1 in hOCT2-HEK cells, open squares and 15.4 µM, log IC_50_ = −4.8 ± 0.1 in hOCT2-MDCK cysts, closed squares, Figure 4B).

**Figure 5:**
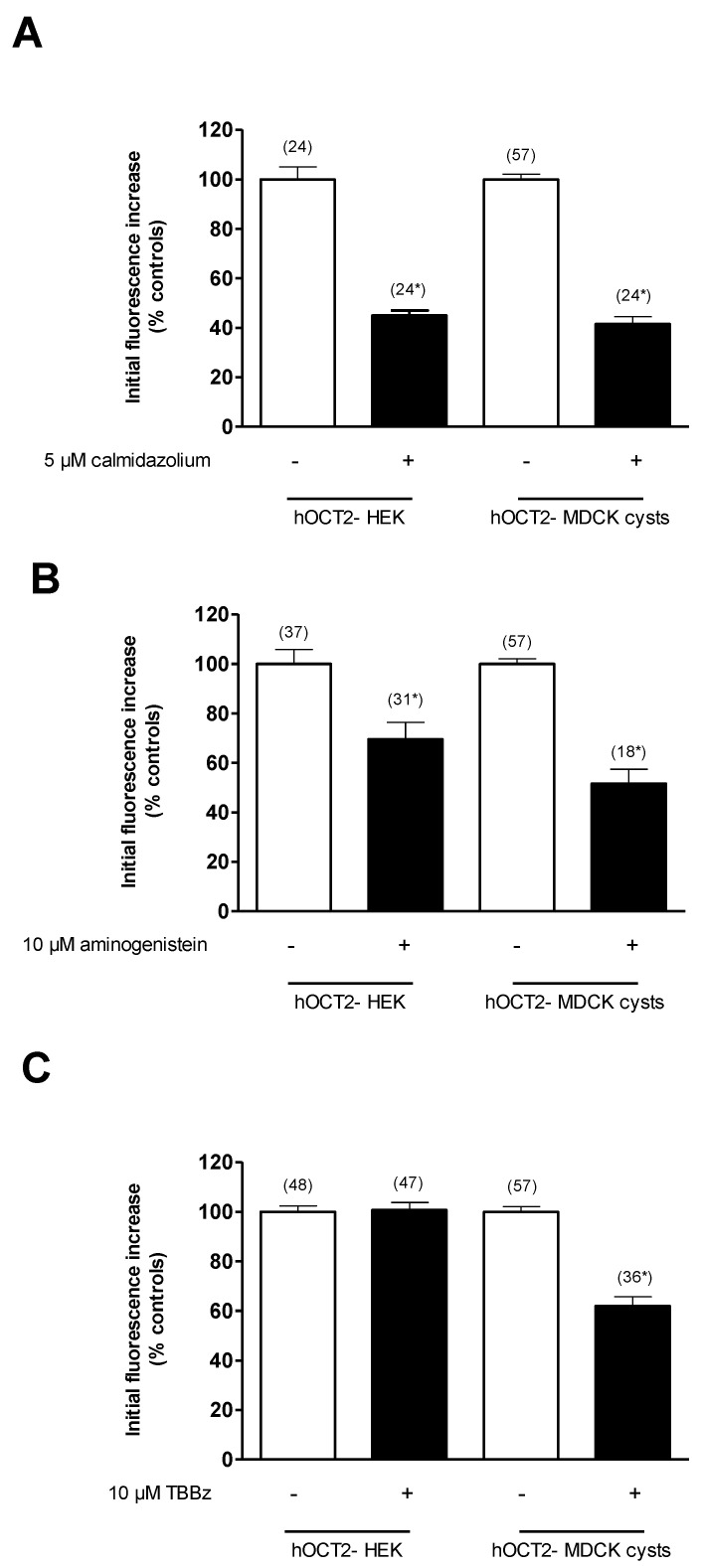
Rapid regulation of ASP^+^ uptake mediated by hOCT2, stably expressed in HEK cells (hOCT2-HEK) or in MDCK cysts (hOCT2-MDCK cysts). The regulation was investigated by inhibiting calmodulin with calmidazolium (5 µM) (**A**), p56*^lck^* tyrosine kinase with aminogenistein (10 µM) (**B**) and CKII with TBBz (10 µM) (**C**). Initial uptake rates of ASP^+^ after 10 min of incubation with these different inhibitors are presented as percentages of the controls. Values are means ± SEM. The numbers on the columns show the number of replicates measured in at least three independent experiments. * A statistically significant difference to the control experiments (*p* < 0.05, unpaired *t*-test).

**Figure 6:**
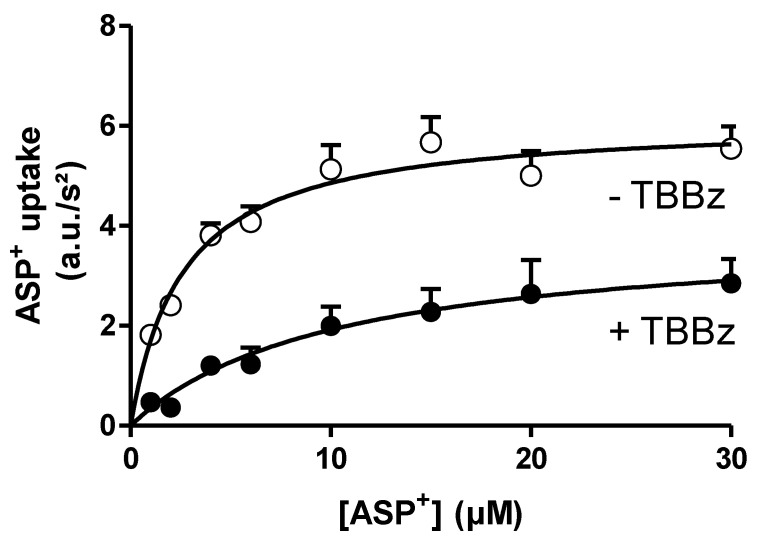
Effect of the inhibition of CKII with 10 µM TBBz on the ASP^+^ transport kinetics measured in hOCT2-MDCK cysts. The open dots show the results of experiments where the cells were preincubated for 10 min with a control solution (-TBBz) before measuring ASP^+^ uptake. Closed symbols represent the results of experiments performed after 10 min of preincubation with 10 µM TBBz (+TBBz). The kinetic parameters were K_m_ = 2.6 ± 0.2 µM and V_max_ = 6.1 ± 0.1 a.u./s^2^ (*N* = 3 with a total of 14–15 replicates for every concentration) for -TBBz and K_m_ = 10.4 ± 4.4 µM and V_max_ = 3.9 ± 0.8 a.u./s^2^ (*N* = 3, with a total of 6–15 replicates for every concentration) for +TBBz. These changes in K_m_ and V_max_ were statistically significantly different (unpaired t-test, *p* < 0.05).

The rapid decrease in ASP^+^ uptake due to the inhibition of Ca^2+^/calmodulin activity by calmidazolium (Figure 5A) and of p56*^lck^* tyrosine kinase by aminogenistein (Figure 5B) showed the same type of acute hOCT2 regulation in hOCT2-HEK cells and hOCT2-MDCK cysts. However, casein kinase II (CKII) inhibition by 4,5,6,7-tetrabromobenzimidazole (TBBz) led to a rapid decrease in ASP^+^ uptake only in the hOCT2-MDCK cysts (Figure 5C), which was associated with significant changes in K_m_ and V_max_. In fact, under CKII inhibition, the affinity of hOCT2 for ASP^+^ in the MDCK cysts decreased from 2.6 ± 0.2 to 10.4 ± 4.4 µM, and the V_max_ decreased from 6.1 ± 0.1 to 3.9 ± 0.8 a.u./s^2^ (Figure 6).

## 3. Discussion

Human organic cation transporter 2 (hOCT2) is highly expressed in the proximal tubules of the kidneys. Here, the transporter localizes to the basolateral domain of the plasma membrane, where it mediates the first step of secretory clearance of organic cations (which are either endogenous substances such as neurotransmitters and metabolism products or exogenous substances such as drugs and xenobiotics). This transport is driven by the electrochemical gradient of the substrates [5]. The secretory clearance of ionic substances by the renal proximal tubules has an important homeostatic role for rapid elimination of endogenous ions and drugs from the body [7]. Therefore, to model the impact of transporters on renal secretory clearance, it is important to know their functional properties. The properties of hOCT2-mediated transport have been extensively studied in cell models such as *Xenopus* oocytes [28] and HEK293 cells [29,30,31]. Even though these expression systems allowed the precise characterization of the functional properties and substrate-binding characteristics of hOCT2, they did not address the functional consequences of the polarized distribution of hOCT2 in the basolateral plasma membrane domain. Few studies addressing this point were performed using freshly isolated human proximal tubules [32]; however, this technique requires access to fresh human samples, which is not always possible. The Madin Darby Kidney (MDCK) cells are a suitable system for the expression of proteins, which have a polarized cellular localization. The MDCK cell line was derived from the kidneys of an adult female cocker spaniel [33], and several clones of these cells exist. The type II MDCK cells (MDCK II), which were used in this study, were isolated from a high-passage parental cell line and displayed low transepithelial resistance values [33]. The MDCK cells are a very important system for studying epithelial polarization [34]. Indeed, there are several works using MDCK cells as the expression system for hOCT2 and other organic cation transporters [35,36,37]. Usually, MDCK cells are cultured on filters to allow distinct experimental access to basolateral and apical membrane domain compartments. However, because of fluorescence quenching or unspecific fluorescence due to the filters, real-time dynamic measurements of transporter function using fluorescent dyes are difficult. In this work, we established a polarized hOCT2 expression system in MDCK cells, which were grown in the presence of extracellular matrix proteins. Under these culture conditions, the cells formed cysts, which were firmly adherent to the matrix and where the basolateral membrane domain was easily accessible for experimental solutions. After having demonstrated the specific expression of hOCT2 in the basolateral membrane domain of MDCK cysts, we showed that the MDCK cysts expressing hOCT2 were able to transport the fluorescent organic cation ASP^+^ and that ASP^+^ uptake was completely suppressed by high concentrations of the inhibitor TPA^+^. A small TPA^+^-sensitive ASP^+^ uptake measured in MDCK cysts expressing the empty vector was probably due to an endogenous expression of canine OCT2, as already demonstrated in [26] and by our PCR and western blot analysis (Supplementary Figure S1). To study whether and how expression in polarized cells influenced the transport properties of hOCT2, the ASP^+^ uptake measured in hOCT2-MDCK cysts was compared to that of HEK293 cells stably expressing hOCT2. The affinity to ASP^+^ and the apparent affinities for TPA^+^ and metformin were the same in both expression systems. However, the apparent affinities to TEA^+^ and baricitinib were significantly different, suggesting that the conformation of the binding sites for these substances was changed by the expression of hOCT2 in a polarized expression system. These findings may be explained by admitting that OCT has a large binding pocket with partially overlapping interaction domains for different substrates [38,39,40]. Indeed, the presence of multiple ligand-binding sites in the OCT is thought to explain the complex interaction of ligands with OCT2, depending on its diverse affinity to substrates with different structures [40,41]. In this optic, only the conformation of the interaction domains for TEA^+^ and baricitinib (but not that of those for TPA^+^ and metformin) may change by expression in a polarized expression system. Similarly, acute regulation of hOCT2 by the inhibition of p56*^lck^* tyrosine kinase or of calmodulin was qualitatively and quantitatively the same in the hOCT2-HEK cell and hOCT2-MDCK cyst models, suggesting that the activity of these kinases is not dependent on cellular polarization. It can be speculated that in both cell systems, p56*^lck^* tyrosine kinase and calmodulin are endogenously active and stimulate hOCT2 function. Indeed, p56*^lck^* has been demonstrated to be present in the plasma membranes of different cell types [42]. Additionally, treatment with calmidazolium has been shown to increase Ca^2+^ mobilization and store-operated Ca^2+^ entry in HEK293 cells [43,44] and to induce rises in the cytosolic Ca^2+^ concentrations in MDCK cells [45]. Conversely, the inhibition of CKII significantly downregulated the transport activity of hOCT2 only in hOCT2-MDCK cysts. It is known that in polarized cells, CKII is present in the plasma membrane, while in non-polarizing cells, it displays mainly a nuclear and cytoplasmatic localization [46]. The membrane localization of CKII is even more prominent in epithelial cells cultivated in an extracellular matrix [46]. Therefore, it can be speculated that the CKII regulates the activity of hOCT2 present on the plasma membrane. Indeed, saturation experiments showed a significant decrease in hOCT2 K_m_ and V_max_ under CKII inhibition, suggesting that the membrane-associated CKII probably may have phosphorylated hOCT2, which is present in the plasma membrane, increasing its activity and prolonging its permanence in this cell compartment. Inhibition of CKII would compromise this regulation.

In conclusion, the hOCT2-MDCK cysts are a suitable model to study the transport properties of hOCT2 in a polarized environment, better reflecting the physiological situation in renal proximal tubules.

## 4. Materials and Methods

### 4.1. Cloning of hOCT2-GFP into the Viral Transduction Vector

The full-length human organic cation transporter 2 (solute carrier (SLC) 22A2, NM 003058) in the expression vector pRc/CMV was kindly provided by H. Koepsell (University of Würzburg), and hOCT2 was amplified from the hOCT2-pRc-CMV and cloned in pEGFP-N3 (Clontech, Saint-Germain-en-Laye, France) via XhoI and BamHI sites (using the forward (For) and reverse (Rev) primers: For-hOCT2 5′-CTC AGA TCT CGA GCT ATG CCC ACC ACC GTG GAC GAT-3′ and Rev-hOCT2 5′-CGG GAT GGA TCC GTT CAA TGG AAT GTC TAG TTT-3′) to obtain a GFP-tagged hOCT2 (hOCT2-GFP) construct. The hOCT2-GFP construct or GFP alone were inserted into the pQCXIH vector (Clontech) via NotI and PacI using the following forward (For) and reverse (Rev) primers for hOCT2-GFP and GFP alone, respectively: For-hOCT2-GFP: 5′-GAT GCG GCC GCA TGC CCA CCA C-3′ and Rev-hOCT2-GFP: 5′-AAG CGG CTT CGG CCA GTA ACG TTA-3′; For-GFP: 5′-GAT GCG GCC GCA TGG TGA GCA AG-3′ and Rev-GFP: 5′-GCT TAA TTA ACT TGT ACA GCT CGT CCA TGC-3′.

### 4.2. Cell Culture

GP2-293 cells (Clontech, Saint-Germain-en-Laye, France) were cultivated in standard DMEM (Biochrom, Berlin, Germany) supplemented with 10% fetal calf serum (FCS, Biochrom) and 1% antibiotics (penicillin/streptomycin, Biochrom).

HEK cells stably expressing hOCT2 were a generous gift from Prof. Koepsell (University of Würzburg, Germany) (for a detailed description of the cells, see [47]). HEK293-hOCT2 cells were grown at 37 °C in 50-mL cell culture flasks (Greiner, Frickenhausen, Germany) in DMEM (Biochrom) containing 3.7 g/L NaHCO_3_, 1.0 g/L D-glucose and 2.0 mM L-glutamine (Biochrom) and gassed with 5% CO_2_. Penicillin (100 U/mL), 100 mg/L streptomycin (Biochrom), 10% FCS and 0.8 mg/mL geneticin (PAA Laboratories, Coelbe, Germany) as the selection antibiotic were added to the medium. Experiments were performed with cells grown to confluence for 3–4 days from passages 12–40.

MDCK cells expressing hOCT2-GFP or GFP alone were cultured in Minimal Essential Medium Eagle (MEME, Sigma/Merck, Darmstadt, Germany) containing 10% FCS, 2 mM L-glutamine and 1% penicillin/streptomycin. For growing MDCK cysts, the wells of a 96-well plate (Greiner) were coated with 2 µL ice-cool Cultrex Basement Membrane Extract (BME2, Trevigen, Gaithersburg, MD, USA), and 100 µL of an MDCK cell suspension (150,000 cells/mL in an MDCK cell culture medium containing 2.5% BME2) was added per well and cultured at 37 °C and 5% CO_2_. At culture day 3, the cell culture medium was renewed. This type of cell culture in an extracellular matrix is referred to as a cyst-forming 3D culture [24]. For this reason, the following MDCK cells expressing hOCT2-GFP or GFP alone and cultured in BME2 will be called hOCT2-MDCK cysts and empty vector (EV) MDCK cysts, respectively. Culture and functional analyses of these cells were approved by the state government’s Landesumweltamt Nordrhein-Westfalen of Essen, Germany (no. 521.-M-1.14/00).

### 4.3. Generation of MDCK Cell Lines Expressing hOCT2-GFP or GFP Alone

The full-length hOCT2 tagged at the carboxy-terminus with GFP (hOCT2-GFP) (The addition of the GFP tag did not change the hOCT2 transport properties; for details, see [48,49].) or the GFP alone (empty vector (EV)) cloned in the expression vector pQCXIH were expressed in MDCK II cells (ECACC 00062107) using a retroviral transduction system, which is known to result in high and stable expression of the transferred constructs, as described previously [50,51,52]. Briefly, a recombinant retrovirus was produced by yjr transfection of GP2-293 cells (Retro-X, Clontech) with a plasmid encoding for the glycoprotein of vesicular stomatitis virus (pVSV-G) and the hOCT2-GFP or GFP alone containing pQCXIH constructs. Next, after filtration of the virus-containing supernatant through a sterile 0.45-μm filter unit (Millipore, Schwalbach am Taunus, Germany), the MDCK cells were infected for 24 h using one volume of fresh Dulbecco’s Modified Eagle Medium (DMEM, Biochrom, Berlin, Germany) and one volume of the virus-containing filtrate supplemented with polybrene (final concentration: 1.5 μg/mL). Thereafter, the virus-containing medium was replaced with a fresh medium, and the cells were regenerated for 24 h. Afterward, the cells were selected by hygromycin treatment (300 μg/mL). GFP-positive cells were isolated with a cell sorter and further cultured. Overexpression of ectopic GFP or GFP-tagged hOCT2 in the stable cell populations was verified by western blot and immunofluorescence analyses (Figure 1).

### 4.4. Western Blot Analysis

For the western blot analysis, MDCK cells grown to confluency were lysed on ice for 20 min with a 300 µL radioimmunoprecipitation assay buffer (RIPA buffer) containing a protease inhibitor cocktail (complete, Merck, Darmstadt, Germany, 1 tablet/10 mL buffer). The lysates were sonicated, resuspended and centrifuged at 10,000× *g* for 5 min at 4 °C. The supernatant was collected and mixed with a NuPAGE^TM^ LDS sample buffer (ThermoFisher Scientific, Oberhausen, Germany) containing 50 mM dithiothreitol (DTT) and then heated at 70 °C for 10 min. After this, equal amounts of protein were given into the wells of the SDS-PAGE gel (Mini-Protean TGX gel, Bio-Rad, Munich, Germany). Electrophoresis was performed for 1 h at 100–140 V. The gel was then blotted for 1 h at 100 V on a polyvinylidene difluoride (PVDF) membrane (Roche Applied Science, Mannheim, Germany). The PVDF membrane was incubated for 1 h in 3% gelatin to block unspecific binding and then overnight with a mouse anti-hOCT2 antibody (kindly provided by Prof. Koepsell [53]) at a 1:250 dilution or with a rabbit anti-GFP antibody (Cell Signaling, Frankfurt am Main, Germany) at a 1:500 dilution. After this, the PDVF membranes were washed and incubated for 1 h with a goat anti-mouse antibody (Dako, Hamburg, Germany) or with a goat anti-rabbit antibody (Dako) coupled with horseradish peroxidase (HRP) at a 1:10,000 dilution and washed again. The immunoreactive bands were detected with an imager system (ChemiDoc™ MP, Bio-Rad, Herkules, USA) by enhanced chemiluminescence using Lumi-Light Plus (Sigma/Merck, Darmstadt, Germany).

### 4.5. Immunofluorescence Analysis

For immunofluorescence analysis, hOCT2-MDCK cysts and EV-MDCK cysts resuspended in a cell culture medium containing 2.5% BME2 were seeded in 8-well Ibidi slides (Ibidi, Gräfelfing, Germany) previously coated with BME2. After 7 days, the cells were washed with PBS, fixed with a 4% paraformaldehyde solution (PFA) for 10 min and then washed again with PBS. F-actin and the nuclei were labeled with Alexa Fluor 594 Phalloidin (Thermo Fischer, 1:200 in 1% BSA) and 4′,6-diamidino-2-phenylindole (DAPI, 1 mg/mL, 1:1000 in 1% BSA), respectively. After 30 min, the cells were washed with PBS and stored at 4 °C in 0.02% sodium azide until evaluation by epifluorescence microscopy (Observer Z1 with Apotome, Zeiss).

### 4.6. Measurement of OCT Function

**Figure 7:**
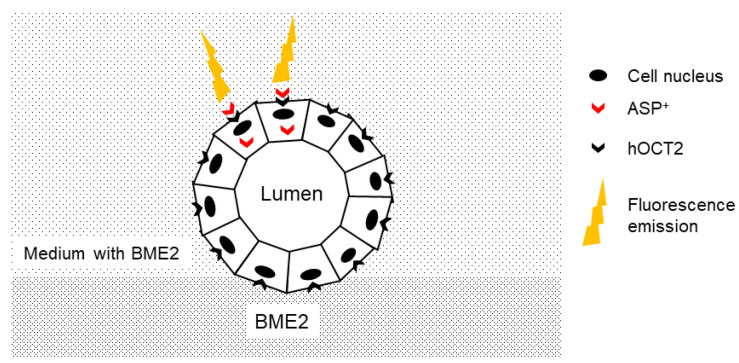
Schematic representation of ASP^+^ uptake experiments in hOCT2-MDCK cysts. The hOCT2-MDCK cells seeded on a BME2 matrix and cultured in a medium containing basement membrane extract (BME2) formed a cyst with a central lumen, and hOCT2 was expressed in the basolateral membrane domain. After ASP^+^ addition, upon binding of ASP^+^ to hOCT2 and uptake into the cells, a fluorescence emission was detected.

The organic cation ASP^+^ was used as a fluorescent substrate of hOCT2 [54]. Since by excitation at 450 nm ASP^+^ displayed an emission spectrum shift from 515 to 580–590 nm upon entering the cells, it was possible to measure its cellular accumulation dynamically with a high time resolution using a microfluorescence plate reader (Infinite F200, Tecan, Männedorf, Switzerland) [55]. To evaluate hOCT2 function, cellular fluorescence was measured before and after ASP^+^ addition in the hOCT2-HEK cells and hOCT2-MDCK and EV-MDCK cysts. Transport measurements were performed at T = 37 °C. Figure 7 shows a schematic representation of the experimental set-up.

Specific hOCT2-mediated ASP^+^ uptake was evaluated by subtraction of the uptake measured in the presence of a high (1 mM) TPA^+^ concentration, a high-affinity hOCT2 inhibitor, from the total uptake.

The affinity of hOCT2 for ASP^+^ was determined by saturation experiments of specific ASP^+^ uptakes determined in the presence of increasing ASP^+^ concentrations (0–20 µM). The apparent hOCT2 affinities (IC_50_) for TEA^+^, TPA^+^, metformin and baricitinib were measured by inhibition of the ASP^+^ uptake (1 and 4 µM ASP^+^ for hOCT2-HEK and hOCT2-MDCK cyst cells, respectively) with increasing concentrations of the competitor (10^−7^–10^−2^ M). In further experiments, acute regulation of the ASP^+^ uptake (1 and 4 µM ASP^+^ for hOCT2-HEK and hOCT2-MDCK cysts, respectively) by short-time incubation (10 min) with 10 µM aminogenistein, 5 µM calmidazolium or 10 µM TBBz as inhibitors of p56*^lck^* tyrosine kinase, Ca^2+^-calmodulin or CKII, respectively, was measured. These inhibitors at these concentrations were chosen because they had already been demonstrated to be effective in regulating hOCT2 activity [37,56]. Solvents (DMSO or ethanol) at the concentration used in the regulation experiments did not change the ASP^+^ uptake (not shown). The effects of the CKII regulation pathway on the kinetic parameters of ASP^+^ uptake in hOCT2- MDCK-3D were investigated, comparing the saturation curves in the presence or absence of the regulator.

### 4.7. Statistical Analysis

The results are presented as the means ± SEM or as box and whisker plots (Min to Max), as indicated in the figure legends. The number of replicates (*n*) measured in at least three independent experiments (*N*) is also shown. Significant differences were calculated using the unpaired Student’s *t*-test or ANOVA with Tukey’s post-test for multiple comparisons as opportune based on *N*. A *p*-value <0.05 was considered statistically significant. Analyses were performed using GraphPad Prism, Version 5.3 (GraphPad Software, San Diego, CA, USA).

## Figures and Tables

**Table 1:** K_m_ ± SEM (only for ASP^+^) and IC_50_ for the inhibition of ASP^+^ uptake by TEA^+^, TPA^+^, metformin and baricitinib (all values given as µM), determined using hOCT2-HEK cells or hOCT2-MDCK cysts. The logIC_50_ values ± SEM and the number of replicates measured in at least three independent experiments are also indicated.

Substances	K_m_ ± SEM or IC_50_ (logIC_50_ ± SEM) in µM and Number of Replicates (*n*) Measured in at Least 3 Independent Experiments	
	hOCT2-HEK	hOCT2-MDCK Cysts
ASP^+^	3.1 ± 0.2 *n* = 14–15	3.6 ± 1.2 *n* = 9
TEA^+^	7.6 (−5.12 ± 0.08) *n* = 24	21.3 * (−4.67 ± 0.11) *n* = 6–12
TPA^+^	1.4 (−5.85 ± 0.11) *n* = 12–30	0.9 (−6.04 ± 0.05) *n* = 18–21
Metformin	804 (−3.10 ± 0.11) *n* = 8–48	884 (−3.05 ± 0.09) *n* = 11–24
Baricitinib	1.1 (−6.00 ± 0.06) *n* = 12–24	15.4 * (−4.81 ± 0.06) *n* = 18–24

* Indicates a statistically significant difference from hOCT2-HEK (*p* < 0.05, unpaired *t*-test).

## Data Availability

Data is contained within the article and supplementary material.

## Supplementary Materials

The following are available online at https://www.mdpi.com/article/10.3390/ijms22179658/s1.
