# Supplementary material for: Properties of Transport Mediated by the Human Organic Cation Transporter 2 Studied in a Polarized Three-Dimensional Epithelial Cell Culture Model
Source: Int J Mol Sci. 2021 Sep 6;22(17):9658. doi: 10.3390/ijms22179658 (PMC8432172; doi:10.3390/ijms22179658)
Supplement: Supplementary file 1 [file ijms-22-09658-s001.zip › ijms-1365873-supplementary.pdf]

Properties of Transport Mediated by the Human Organic Cation Transporter 2 Studied in a Polarized Three-Dimensional Epithelial Cell Culture Model

Tim N Koepp †, Alexander Tokaj †, Pavel I Nedvetsky, Ana Carolina Conchon Costa, Beatrice Snieder, Rita Schröter and Giuliano Ciarimboli *

Medicine Clinic D, Experimental Nephrology, University Hospital of Münster, 48149 Münster, Germany; tim.koepp123@gmail.com (T.N.K.); alex.tokaj@uni-muenster.de (A.T.); nedvetsky@uni-muenster.de (P.I.N.); carolconchon@gmail.com (A.C.C.C.); bea.snieder@gmail.com (B.S.); ritas@uni-muenster.de (R.S.)

* Correspondence: gciari@uni-muenster.de; Tel.: +49-251-83-56981

† These authors equally contributed to the manuscript

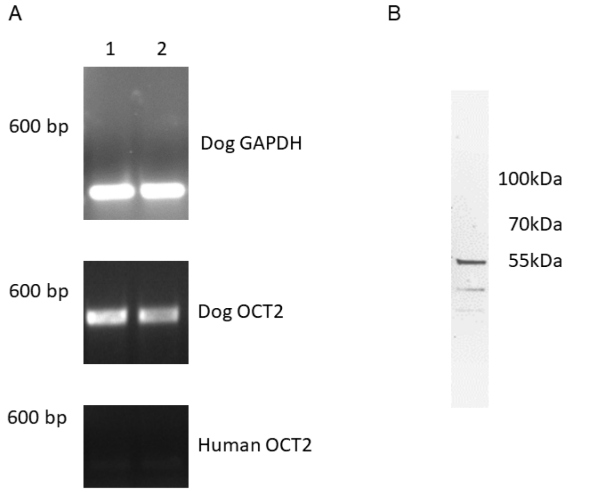

Supplentary data, Figure S1. OCT2 endogenous expression in empty vector (EV)-MDCK cells. Panel **A** shows a PCR analysis of two independent (1 and 2) EV-MDCK cells samples for canine GAPDH and OCT2 expression. The primers (forward, F, and reverse, R) used were (in 5′ to 3′ direction) F: CCCACTCTTCCACCTTCGAC and R: CCTTGGAGGCCATGTAGACC for canine GAPDH and F: GTTGGGCGGAGATATCGGAG and R: AAGGCCCATGTGCATGATGA for canine OCT2. Amplification using specific primers for human OCT2 (F: CGCCATTCCTGGTCTACCGGC and R: GCTTCCTCGATGGTCTCAGGC) did not give any product. The height of the 600 basepairs (bp) ladder is also shown. Panel **B** shows a Western blot analysis of endogenous OCT2 expression in lysates from EV-MDCK cysts performed as described in Material and Methods, using an antibody against hOCT2 (Sigma/Merck, Darmstadt, Germany) at a 1:500 dilution. Cells expressing EV display one band at 55 kDa, probably corresponding to endogenous OCT2 expression. Two smaller bands, probably corresponding to degradation products are also evident. Performing the Western blot analysis using the hOCT2 antibody as in Figure 1 of the manuscript did not show any signal, probably because of the high specificity of that antibody.
